# A review of issues of nomenclature and taxonomy of *Hypericum perforatum* L. and Kew's Medicinal Plant Names Services

**DOI:** 10.1111/jphp.12831

**Published:** 2017-10-16

**Authors:** Elizabeth Anne Dauncey, Jason Thomas Whitley Irving, Robert Allkin

**Affiliations:** ^1^ Royal Botanic Gardens, Kew Richmond Surrey UK

**Keywords:** *Hypericum perforatum* L., Identification of Medicinal Products (IDMP), Medicinal Plant Names Services (MPNS), nomenclature, St John's wort, taxonomy

## Abstract

**Objectives:**

To review which names are used to refer to *Hypericum perforatum* L. in health regulation and medicinal plant references, and the potential for ambiguity or imprecision.

**Key findings:**

Structured searches of Kew's Medicinal Plant Names Services Resource, supplemented with other online bibliographic resources, found that the scientific name *Hypericum perforatum* L. is used consistently in the literature, but variation between subspecies is rarely considered by researchers. Research is still published using only the common name ‘St John's wort’ despite it being imprecise; at least 80 other common names are also used for this plant in multiple languages.

**Summary:**

Ambiguous and alternative plant names can lead to ineffective regulation, misinterpretation of literature, substitution of raw material or the failure to locate all published research. Kew's Medicinal Plant Names Services (MPNS) maps all names used for each plant in medicinal plant references onto current taxonomy, thereby providing for disambiguation and comprehensive access to the regulations and references that cite that plant, regardless of the name used. MPNS also supplies the controlled vocabulary for plant names now required for compliance with a new standard (Identification of Medicinal Products, IDMP) adopted by medicines regulators worldwide.

## Introduction

*Hypericum perforatum* L. is a widely researched and traded medicinal plant (see other articles in this issue), indicated, among many other conditions, for mild depression in Europe and inflammation in East Asia.^1^, ^2^, ^3^, ^4^ It has been studied extensively by researchers interested in the pharmacological properties, particularly to explain its use in treating depression. However, accessing and interpreting the results of this research can be frustrated by the inappropriate and imprecise use of plant names and inappropriate use of botanical classification by researchers.

The interrelated problems of nomenclature (names) and taxonomy (classification) are relevant to all plants investigated for their medicinal properties. It is important for natural product researchers working on a plant to be aware of all the names that have been used for it, and which are potentially ambiguous, so that they can undertake effective and comprehensive literature searches and data mining. In addition, to maximise the impact of their own publications, they should also be aware of and include the names that will ensure their research will be discovered.^5^

This paper draws on (1) published research, (2) the experience of building a plant names resource for Kew's Medicinal Plant Names Services (MPNS) and (3) the review of manuscripts submitted for publication to natural product journals, to provide a comprehensive outline of the issues associated with the use of scientific and non‐scientific plant names in research on medicinal plants.

To examine how far these nomenclatural issues apply to *H. perforatum,* it was first necessary to determine the current understanding of the classification of this species from published revisions and online resources. *Hypericum perforatum* is a morphologically variable species, and this variation is recognised as four subspecies. The natural distributions of the species and its subspecies, and the other species of *Hypericum* to which it is most closely related, are also presented. These are important considerations for natural product researchers wishing to ensure the accurate identification of their source plant material, reviewing the reliability of published research, or considering alternative plants to study.

The MPNS Resource is briefly described below, and its features and limitations explored. The Resource was used, supplemented by limited searches of other online bibliographic resources, to find the scientific (accepted names of species and subspecies, and synonyms), and non‐scientific names as they are actually employed in regulations, references and research relating to medicinal uses of *H. perforatum*. This paper is significant as it is the first to review which scientific and non‐scientific names are used for *H. perforatum* in medicines regulations and references, to present the names issues that are relevant to this species and discuss the implications for finding, carrying out and interpreting pharmacological research into this and other plants.

## Kew's Medicinal Plant Names Services Resource

The Royal Botanic Gardens, Kew, manages a number of the globally significant taxonomic and nomenclatural resources for flowering plants.^6^, ^7^, ^8^, ^9^ MPNS, which was developed with support from the Wellcome Trust, has made these data more accessible to respond to the identified needs of those researching, regulating and using medicinal plants. The Resource was designed to help ensure unambiguous communication in recognition of the many challenges, outlined in section Problems with Plant Names and Their Relevance to *Hypericum perforatum*, that using plant names poses for these groups.

A unique feature of the MPNS Resource is that it links Kew's authoritative, peer‐reviewed taxonomy to the scientific and non‐scientific names used for medicinal plants, exactly as they are spelt in pharmacopoeias, and other relevant publications. This enables researchers to use the name that they are familiar with to access the taxonomy and nomenclature for the plant that they are studying and alerts them to possible ambiguities and alternative synonyms. This paper examines the usefulness of MPNS as a tool to explore potential issues with nomenclature and taxonomy of medicinal plants, using *H. perforatum* as an example to complement the other articles in this special issue of the JPP.

The MPNS Resource is publically available to search through the portal and used to provide a range of services for organisations managing medicinal plant information. The results presented in this paper are based on Version 6 of the MPNS Resource accessed via the portal.^6^, ^10^ For details of the content of the MPNS Resource Version 6, see Table S1 available online.

## The Genus *Hypericum* and the Position of *H. perforatum* Within It

The genus *Hypericum* is distributed globally, although generally absent from environments that are subject to extreme heat, cold, dryness or moisture.^11^ The genus is currently considered to include over 500 species divided between two subgenera; one mainly Old World in which dark hypericin‐containing glands are present, and the other mainly New World in which such glands are absent.^12^ It can be further divided, using differences in morphological characters, into 36 sections. Robson's multipart monograph of the genus was published between 1977 and 2015 and used traditional morphogeographical methods to discern morphological trends and determine their evolutionary direction. Other papers provide overviews,^11^, ^13^ consider specific aspects such as molecular phylogenetics and morphological evolution^14^, ^15^ or summarise the various treatments and discuss the differences between them.^12^

A full systematic treatment of *Hypericum* section *Hypericum*, subsection *Hypericum*, series *Hypericum,* is provided by Robson.^16^ *Hypericum perforatum* is placed in series *Hypericum*, along with the 10 species of *Hypericum* to which it is most closely related. The 11 species in this series are divided into one group of four species centred in Europe and the Mediterranean, and a group of six species which is centred in north‐east Asia, but also has one species that is confined to western North America and another that has spread to westward into Europe. The remaining species in series *Hypericum* is *H. perforatum*, which is morphologically and geographically intermediate between *Hypericum maculatum* Crantz from the first group and *Hypericum attenuatum* Fisch. ex Choisy from the second group. *Hypericum perforatum* has a wide natural distribution from the Azores via Serbia to China (south to Yunnan) and via the Mediterranean region to the western Himalayas. It has also been introduced into many other parts of the world.^11^

Robson^11^, ^16^ postulates that *H. perforatum* is likely to be an allotetraploid (2*n* = 32) derivative of a cross between *H. maculatum* subsp. *immaculatum* (Murb.) A.Fröhl. and *H. attenuatum*, both diploid (2*n* = 16), that occurred where the distributions of these two species overlapped in the Altai region of Siberia. Their distributions still almost overlap in western Siberia, where *H. maculatum* is represented by subsp. *maculatum*. Although the subspecies that is morphologically closest to *H. perforatum*,* H. maculatum* subsp. *immaculatum* is now restricted to the central Balkans, its distribution is likely to have once included western or even central Siberia. *H. attenuatum* occurs from western Siberia to China. *H. perforatum* is known to form hybrids with several species of *Hypericum* including *H. maculatum*, and these hybrids can be morphologically indistinguishable from *H. perforatum*.

## *Hypericum perforatum* L. and Its Subspecies

### Overview

Kew's taxonomy represented within the MPNS Resource recognises *H. perforatum* L. as an accepted name with four accepted subspecies within it, which follows Robson^16^ who opted to recognise these four subspecies for practical reasons. As a general rule, rather than an absolute requirement, subspecies form distinct populations with some form of separation, such as in their distribution or flowering time, and have minor morphological differences. The observed variation in the morphological characters of the subspecies of *H. perforatum*, however, is more or less continuous, and the subspecies serve to define the differences that occur across the distribution of the species. The subspecies are:

1. *Hypericum perforatum* subsp. *perforatum*

Differentiating characters: Leaves usually shortly petiolate (to 1 mm), herbaceous, concolourous (not glaucous beneath), usually oblong to ovate or elliptic, the base narrowed (cuneate), but in Russia and Scandinavia, leaves are sessile, the base rounded; inflorescences not usually congested, branches relatively short, straight; petal laminar glands all pale to mostly black but some always pale.

Distribution: Found from northern and north‐western Europe to the west to central Siberia to the east. After a break in occurrence, it recurs almost unchanged in China to the south‐east of the Mongolian desert and steppe region; moving south‐eastward it transforms gradually into subsp. *chinense*.

- *H. perforatum* subsp. *chinense* N.Robson

Differentiating characters: Leaves shortly petiolate (to 1 mm), narrow; inflorescences congested, branches relatively long, curved‐ascending; petal laminar glands all black or absent.

Distribution: China (eastern Qinghai and Gansu east to Shandong and south to Yunnan and Guizhou).

- *Hypericum perforatum* subsp. *songaricum* (Ledeb. ex Rchb.) N.Robson

Differentiating characters: Leaves sessile, often subcoriaceous (somewhat leathery) and discolourous, oblong to oblong‐ovate, base more or less shallowly cordate‐amplexicaule; sepals finely acuminate; petal laminar glands usually all pale; capsule valves with lateral vitae linear or slightly swollen overall, not interrupted.

Distribution: In the mountains of Kazakhstan and Kyrgyzstan, and Xinjiang, China. After a gap of 2500 km, it recurs in southern Russia and Krym, where after a short transitional zone it is replaced by subsp. *veronense*.

- *Hypericum perforatum* subsp. *veronense* (Schrank) H.Lindb., which includes var. *angustifolium* DC. and var. *microphyllum* DC. as synonyms

Differentiating characters: Leaves sessile, at least on main stem, usually narrowly triangular‐lanceolate to linear or, if broader, then short (*c*. 5–10 mm long), base rounded to cuneate; sepals acute; petal laminar glands usually all pale; capsule valves with lateral vittae swollen at base or interrupted to short and irregular (vesicular).

Distribution: From Turkey westward into southern Europe, the Mediterranean and Macaronesia, south to Saudi Arabia and the Sudan.

### Recognition of *Hypericum perforatum* subspecies in published research and regulations

None of the references in the MPNS Resource refers to any of the subspecies, referring only to the species as a whole. A search of PubMed returned less than ten references for each search using a particular infraspecific name (see Table 1). It is important to underline that there are limitations to the search potential of PubMed when searching plant names, due to the way search strings are broken into component terms and then matched to a variety of NCBI ontologies. As a result, not all papers referring to a particular subspecies will be detected by a search using its name. The return can be maximised by omitting the infraspecific status, for example a search using ‘*H. perforatum angustifolium*’ returns nine references whereas including ‘subsp.’ in the string reduces this to five. Including ‘ssp.’ in the string means that no references are found (searches carried out on 10/10/2016).^17^ Regardless, it is evident that most published research into *H. perforatum* fails to identify which subspecies is under study (to which subspecies the plant material being investigated belongs), or whether it might be a mixture.

**Table 1:** Number of references returned for selected search terms relating to the infraspecific taxa for *Hypericum perforatum*

Search term; note that ‘subsp.’ is not included in the search string	No. refs returned	No. refs relevant to infraspecies
‘*Hypericum perforatum chinense*’	14	0
‘*Hypericum perforatum songaricum*’	0	0
‘*Hypericum perforatum veronense*’	4	4
‘*Hypericum perforatum angustifolium*’	9	9

## Problems with Plant Names and Their Relevance to *Hypericum perforatum*

Reviewing manuscripts submitted for publication in natural product journals enabled a compilation by the present authors of issues routinely found with plant names in manuscripts, previously published as part of an editorial which advised authors on best practice regarding the use of scientific plant names.^5^ Although such problems are frequently picked up at the review stage, leading to rejection of the manuscript or a requirement to revise, a significant number can still be found in published research.^18^, ^19^ Collating the plant names used in medicinal plant regulations and research for the MPNS Resource highlighted further problems, and these have been supplemented by a review of published discussions, including,^20^, ^21^, ^22^, ^23^ to present a comprehensive outline of the issues associated with the use of scientific and non‐scientific plant names in research on medicinal plants. The implications of each issue and their relevance to *H. perforatum* as an example are described in turn.

### Scientific names

Scientific names are also known as botanical or Latin names. For species, the term ‘binomial’ is also used. They are scientific because, unlike any other class of name referred to in this paper, they are formally described and published, according to the rules of the International Code of Nomenclature.^24^ Formal publication includes designation of one or more specimens, known as a ‘type’, that fix the meaning of the name for all time and, in the case of doubt, the physical specimen can be referred to resolve disputes or provide DNA or samples for chemical or anatomical analysis. For further information, see Refs 25, 26.

#### Relevance to *Hypericum perforatum*

Carl Linnaeus published the name *H. perforatum*, in his *Species Plantarum* in 1753,^27^ and his authorship is indicated by an ‘L.’ following the binomial. Linnaeus gave the provenance of *H. perforatum* as ‘Habitat in Europæ pratis’, which is probably Sweden. Robson^28^ selected a specimen in Linnaeus's herbarium (Herb. Linn. 943/34; originally as 943/94 in error) as the type specimen for the name [the specimen can be viewed at http://www.nhm.ac.uk/our-science/data/linnaean-typification/search/detailimage.dsml?ID=458700].

#### Issue 1: Synonyms

One plant may be referred to using several different scientific names (synonyms). Analysis of Version 6 of the MPNS resource has shown that medicinal plants have on average nine synonyms, with 93 species having 100 or more synonyms. 71% of plants are referred to using only their currently accepted name in the references captured. 11% of the plants in the MPNS Resource, however, are referred to across these references by both their accepted name and one or more of their synonyms. The remaining 17% of the plants in the Resource are referred to using only one or more of their synonyms (i.e. the current accepted name was not used in any of the references).

##### Implications

The publication of information under more than one scientific name creates confusion and makes finding all published research more difficult. Unless a literature search uses all possible names, it is likely to miss a proportion of the published research. Consequently, research may be unintentionally repeated, and may ultimately not be published as it is not novel, resulting in wasted time and resources. In addition, a disregard or lack of awareness of synonymy can result in the same plant being included in a publication or regulation more than once under different names, with the potential for inconsistency in the presented results and incorrect conclusions.

##### Relevance to *Hypericum perforatum*

*Hypericum perforatum* is one of the 71% of plants in the MPNS Resource that is referred to by the accepted scientific name only throughout the references included in the Resource. Despite four subspecies being recognised for *H. perforatum*, all references in the MPNS Resource that include this plant refer to it at the species level. The review of synonyms was restricted to the type subspecies, *H. perforatum* subsp. *perforatum*, which has 21 scientific synonyms, nine of which are at the species level and the remainder of which are infraspecific names (varieties, formas and a subvariety); the other subspecies had far fewer synonyms. A search in the NCBI's PubMed for the nine synonyms at the species level found no references, whereas a search for ‘*H. perforatum*’ returned 1101 entries.^17^ This, and the references in the MPNS Resource, demonstrates the consistency with which medical research literature uses the name ‘*H. perforatum*’ for this species.

A further species of *Hypericum* that is recorded in the MPNS Resource as having a medicinal use, *Hypericum brasiliense* Choisy, illustrates why out‐of‐date taxonomy is a problem. The MPNS Resource indicates that the species has been recorded from three references. One reference^29^ refers to this species using the currently accepted scientific name, but the other two use a synonym, *Hypericum laxiusculum* A.St.‐Hil. (with the author spelt variously as ‘A.Saint‐Hil.’ or as ‘St.Hill.’).^30^, ^31^ If a search for research published on this species is then made in PubMed using this synonym ‘*H. laxiusculum*’, no matches are found, whereas a similar search using the accepted name ‘*H. brasiliense*’ finds eight references. A researcher who is only aware of the synonym *H. laxiusculum* will think that PubMed does not include publication citations of relevance to this species. The MPNS Search portal enables users to simultaneously search PubMed using all known scientific synonyms of each plant guaranteeing success in finding all PubMed records relevant to that plant.

#### Issue 2: Plant name author citations

Around 4% of scientific binomials (genus + species) are ‘homonyms’, that is, the same name has been applied inadvertently, usually by different authors, to different species.^32^ Where such homonyms exist, it is not possible to know which of the species is being referred to unless the author citation is included. In addition, there are numerous instances of errors in author citation leaving the identity of the plant in question. It is therefore good practice to include the author, as cited in an authoritative taxonomic resource, as an integral part of the plant name.

##### Implications

If care is not taken when citing the author for a name, and the wrong author is included, research results will be associated with the wrong plant or at least interpreted incorrectly. Omission of the author leaves the intended species in question if homonyms exist. The results can then be attributed to the wrong species, or they may be dismissed as unreliable.

##### Relevance to *Hypericum perforatum*

Linnaeus is the only person to have published this binomial, so the potential problem of homonyms is not relevant for this species. It is still good practice to include the author citation ‘L.’ the first time the binomial is used in a paper.

Homonyms do, however, exist for other *Hypericum* binomials. *Hypericum maculatum* Crantz, for example, was published in 1763 and is an accepted name, whereas *H. maculatum* Walter was published later, in 1788, and is considered to be a synonym of *H. punctatum* Lam. Unless the author is included after the binomial *H. maculatum,* it is not clear which of these medicinal species is being referred to. Searching the MPNS portal with the name ‘*H. maculatum*’ returns five plants recorded as having a medicinal use, revealing further complexity: the two already mentioned, the two accepted subspecies of *H. maculatum* Crantz and a third species *H. tetrapterum* Fr. which has a synonym *H. maculatum* subsp. *quadrangulum* (L.) Hayek.

#### Issue 3: Incorrectly spelt names

Variations in the spellings of scientific names exist in botanical and taxonomic literature and resources, for reasons associated with applying the botanical code. This is the case, however, for relatively few names. By far, the greatest source of variations in spelling is simple errors in the literature. This is often the result of inadequate proof reading and an unfamiliarity with scientific plant names. Such names are at best ambiguous and at worst meaningless.

##### Implications

Research using incorrectly spelt scientific names will often not be found during literature searches. This will result in a loss of potential citations and therefore credit. The use of such names can result in loss of credibility and reputational risk.

##### Relevance to *Hypericum perforatum*

The 37 different references in the MPNS Resource all spell the binomial correctly, ‘*H. perforatum*’, and usually include the author, spelt ‘L’, ‘L.’ or ‘Linné’. Variations in spelling of the scientific name can be found in published research, for example one paper on *H. perforatum* 33 spelt the genus as ‘*Hypericum*’, ‘*Hipericum*’ and ‘*Hyperycum*’. Such instances are, however, rare.

### Non‐scientific names

The MPNS Resource contains 68 772 different non‐scientific names for 18 500 plants. This sample of the almost endless number of non‐scientific names in use for plants is useful in highlighting current or possible confusion concerning the species to which a particular name refers. The MPNS Resource only includes references that use scientific as well as non‐scientific names for plants, but a significant amount of research is published without using scientific names at all.^5^, ^19^ This section reviews the implications of using only non‐scientific names in research, with reference to the names in the MPNS Resource, compared with those in PubMed and Google Scholar.

Among the publications included in the MPNS Resource that refer to *H. perforatum* are a number of pharmacopoeias, such as the British, European, Chinese, Argentinian, US and US Homeopathic Pharmacopoeias. The plant is also cited in the European Medicines Agency (EMA) Community Herbal Monographs and Herbs of Commerce and is mentioned in regional and global books on medicinal plant use and ethnobotanical studies. (See Table S2 for a list of all references in the MPNS Resource V6 that refer to *H. perforatum*). The MPNS Resource includes all the diverse pharmaceutical and common names (including drug and trade names – see below for definitions) that have been used for *H. perforatum* among these regulations and other medicinal plant references in multiple languages.

As the issues associated with the use of the various classes of non‐scientific name can overlap, they are summarised here as simply as possible: *Non‐specificity* – one non‐scientific name will often have been used to refer to several different plant species; *Non‐universality* – different publications and people will use the same name for different plants, and different names for the same plant; *Instability* – the meaning of a non‐scientific name can vary over time, for example over different generations of a community or between different editions of a pharmacopoeia; and *Lack of formality and standardisation* – while pharmacopoeias do include definitions of the drug or substance that each pharmaceutical or drug name refers to, these names are meaningless unless the particular pharmacopoeia (including the edition) is also specified. Common names are completely unregulated, the exception being the standard common names described below.^34^, ^35^

#### Relevance to *Hypericum perforatum*

Despite the lack of precision and scientific rigour, non‐scientific names are still used in research publications without reference to the scientific name of the plant studied. The most commonly used non‐scientific name in English for *H. perforatum* is ‘St John's wort’ (Table S3). A search of PubMed retrieved more articles using the common name than the scientific name, while the reverse was true for Google Scholar (Table 2). A comparative analysis of the content of these papers is beyond the scope of this paper, but investigation of a random sample of the papers citing the common name demonstrated that research is being published without use of the scientific name anywhere in the paper. Regardless of the scientific merit in the findings of such papers, their usefulness is reduced if the species studied is not made completely clear.

**Table 2:** Comparative searches of PubMed and Google Scholar

Search PubMed for ‘St John's wort’ = 1445 results
Search Google Scholar for ‘St John's wort’ = 25 800 results
Search PubMed for ‘*Hypericum perforatum*’ = 1113 results
Search Google Scholar ‘*Hypericum perforatum’ *= 38 600 results
Search performed on 31/10/2016

Non‐scientific names used for medicinal plants can be divided into several classes: common, pharmaceutical, drug and trade names. These are briefly outlined followed by the relevance of each class to *H. perforatum*. While the potential for confusion with pharmaceutical and drug names is more restricted than with common names, several studies have highlighted the imprecision of using them.^20^, ^21^, ^36^

### *Pharmaceutical names*

also known as pharmacopoeia names and sometimes as Latin or Latin genitive names: These are used as monograph titles in some pharmacopoeias. They are Latinised and normally consist of a genus name and a plant part name. Farah *et al*.^20^ explore how the use of the genus name alone to form pharmaceutical names can cause confusion when interpreted as referring to any species from that genus. Although they are Latinised in the same way that scientific names are, they are not formally published and regulated in the same way as botanical scientific names. Their meaning is defined by pharmacopoeias; however, the definition of the same pharmaceutical name can differ between different pharmacopoeias and even between different editions of the same pharmacopoeia, and different pharmacopoeias can use different pharmaceutical names for the same plant. While scientific names always refer to a single plant, pharmaceutical names can include one or more species in their definition.

#### Relevance to *Hypericum perforatum*

The MPNS Resource includes six pharmaceutical names for *H. perforatum* (Table 3). It shows that different pharmacopoeias use different pharmaceutical names for essentially the same part of the plant: the European Pharmacopoeia^37^ uses ‘Hyperici Herba’ for the flowering top, while the Chinese Pharmacopoeia^38^ uses ‘Hyperici Perforati Herba’ for aerial parts; variations on these, that is ‘Herba hyperici’ and ‘Herba Hyperici Perforati’, have also been used in other references. One article, *Medicinal Plants of the Russian Pharmacopoeia*,^39^ which contextualises and summarises the definitions of herbal medicines defined in the Russian Pharmacopoeia, is an example of a pharmaceutical name that includes more than one species in its definition. ‘Herba hyperici’ is defined as ‘*H. perforatum* L., *H. maculatum* Crantz’; presumably, in the Russian Pharmacopoeia; this would be an ‘either/or’ or and ‘and/or’ definition.

**Table 3:** Pharmaceutical names linked to *Hypericum perforatum* in the references in the MPNS resource V6

Pharmaceutical name	Part of plant used medicinally	Reference abbreviation
Herba hyperici		Med. Pl. of the Russian Pharm. (Shikov, 2014)
Herba Hyperici	Flower, aerial parts	WHO Monographs Med. Pl. 2 (2004)
Herba Hyperici Perforati	Aerial parts	Pharmacopoeia of China (2005)
Hyperici herba	Herba	EMA Community Monographs (2006–2014)
Hyperici herba	Herba	EMA Community Monographs (2006–2014)
Hyperici herba	Flower	European Pharmacopoeia, 6th edn. (2007)
Hyperici herba	Flowering top	European Pharmacopoeia, 7th edn. (2012)
Hyperici herba	Herb	Med. Pl. of the World (Wyk & Wink, 2004)
Hyperici Perforati Herba	Aerial parts	Pharmacopoeia of China (2010)
Hyperici Perforati Herba		Pharmacopoeia of China (2015)
Hyperici summitates cum floribus recentes	Stem tips	Pharmacopoeia Helvetica (Swissmedic, 2006)
*Hypericum perforatum* ad praeparationes homoeopathicas	Flowering plant	European Pharmacopoeia, 6th edn. (2007)
*Hypericum perforatum* ad praeparationes homoeopathicas	Whole plant	European Pharmacopoeia, 7th edn. (2012)

### Drug names

As with pharmaceutical names, these are used as monograph titles in pharmacopoeias and often include a plant part; however, they are written in the native language rather than Latinised. The drug names of Chinese *materia medica* are written in Chinese characters and also provided as pin yin transliterations.^36^

### Relevance to *Hypericum perforatum*

The MPNS Resource does not include the category ‘drug name’ and any drug names in the references that it covers these are grouped with common names.

## *Common names*

also known as vernacular names, or by the country or language (e.g. English name, French name.): They can be included in monographs in addition to the names used in the titles and are also used in popular and less official texts. Common names can be written in any language. Herbs of Commerce, published by the American Herbal Products Association (AHPA), categorises some names as ‘Standard Common Names’; those in the first edition^34^ have a regulatory status in the United States, although those in the second edition^35^ are now widely adopted. Nonetheless, some of these ‘standardised’ names vary from one edition to the next as practice evolves within the industry. Common names in general, however, are unregulated and non‐specific.

### Relevance to *Hypericum perforatum*

‘St John's Wort’ is used in common parlance in the English language to mean any species of the genus *Hypericum*; potentially all 500 species. Each species will have a different chemistry. The use of the name ‘St John's Wort’ dates back to when the pagan feast to celebrate Midsummer was Christianised and dedicated to St John the Baptist. *Hypericum* plants, believed to have the power to ward off evil spirits, were used to decorate religious images on Midsummer's Eve. In the Aegean, *H. empetrifolium* Willd. was used for this purpose, but elsewhere in Europe, it was *H. perforatum*, the most common species in that region.^11^

A search of the MPNS portal (Version 6) with ‘St John's Wort’ returns eight different possible plants. All are species of *Hypericum* (see Table 4). Other than *H. perforatum*, each of the species is only cited in one reference. For six species, the name St John's Wort is qualified with an adjective: thus, for example, *Hypericum ellipticum* Hook. is recorded as ‘Pale St. John's Wort’. The unqualified name ‘St John's Wort’ is usually recorded in MPNS as being used for *H. perforatum*; however, one reference^48^ cites this name for a different species, *Hypericum monogynum* L. *Hypericum perforatum* is also referred to as ‘Common St John's Wort’ and ‘Perforate St John's Wort’ in a number of references.

**Table 4:** Species in the MPNS resource that match the search term ‘St John's Wort’. See Table S1 for full details of the references in the Reference abbreviation column

Scientific name in taxonomic resource	Non‐scientific name in reference	Reference abbreviation
*Hypericum ascyron* L.	Great St. John's Wort	Native American Ethnobotany (Moerman, 1998)
*Hypericum ellipticum* Hook.	Pale St. John's Wort	Native American Ethnobotany (Moerman, 1998)
*Hypericum fasciculatum* Lam.	Peelback St. John's Wort	Native American Ethnobotany (Moerman, 1998)
*Hypericum japonicum* Thunb.	Matted St. John's wort	GRIN Report: World Economic Plants (Wiersema, 1999)
*Hypericum monogynum* L.	St John's wort	Med. Pl. Indian Ocean Ils. (Gurib‐Fakim *et al*., 2004)
*Hypericum perforatum* L.	Common St John's Wort	WHO Monographs Med. Pl. 2 (2004)
*Hypericum perforatum* L.	Common St. John's Wort	Native American Ethnobotany (Moerman, 1998)
*Hypericum perforatum* L.	Common St. John's wort Herb	Pharmacopoeia of China (2010)
*Hypericum perforatum* L.	Perforate St John's wort	WHO Monographs Med. Pl. 2 (2004)
*Hypericum perforatum* L.	Perforate St. John's wort	Med. Pl. of the World (Wyk & Wink, 2004)
*Hypericum perforatum* L.	Perforate St. John's wort	GRIN Report: World Economic Plants (Wiersema, 1999)
*Hypericum perforatum* L.	St John's Wort	WHO Monographs Med. Pl. 2 (2004)
*Hypericum perforatum* L.	St. John's Wort	British Pharmacopoeia (2011)
*Hypericum perforatum* L.	St. John's wort	Med. Pl. of the World (Wyk & Wink, 2004)
*Hypericum perforatum* L.	St. John's Wort	British Pharmacopoeia (2008)
*Hypericum perforatum* L.	St. John's wort	EMA Community Monographs (2006–2014)
*Hypericum perforatum* L.	St. John's wort	European Pharmacopoeia, 6th edn. (2007)
*Hypericum perforatum* L.	St. John's Wort	British Pharmacopoeia (2014)
*Hypericum perforatum* L.	St. John's Wort	U.S. Pharmacopoeia USP 32 (2008)
*Hypericum perforatum* L.	St. John's wort	GRIN Report: World Economic Plants (Wiersema, 1999)
*Hypericum perforatum* L.	St. John's wort	Herbs of Commerce (McGuffin *et al*., 2000)
*Hypericum perforatum* L.	St. John's Wort	U.S. Pharmacopoeia USP 37 (2013)
*Hypericum perforatum* L.	St. John's wort	European Pharmacopoeia, 7th edn. (2012)
*Hypericum punctatum* Lam.	Spotted St. John's Wort	Native American Ethnobotany (Moerman, 1998)
*Hypericum scouleri* Hook.	Scouler's St. John's Wort	Native American Ethnobotany (Moerman, 1998)

In addition to St John's Wort and variations on this name, *H. perforatum* is also known by numerous other non‐scientific names around the world. There are 82 unique non‐scientific names for this single species in the MPNS Resource, excluding pharmaceutical names (See Table S3). Many of these names share their origin with St John's Wort, such as ‘erba di San Giovanni’, ‘hierba de San Juan’ and ‘Johanniskraut’, and others refer to the perforate leaves, for example ‘millepertuis’. The origin of others is more obscure: ‘Lord God's wonder plant’ and ‘Tipton weed’ being two of many.

## Trade names

This is the least well‐defined class of name. Trade names are used in commerce, to refer to both raw materials as they are traded along the supply chain and to the final manufactured products. They can also be considered to be the ‘ingredients’ listed on such products as well as the name of the product itself. Trade names can be any of the other classes of non‐scientific name, that is pharmaceutical, drug and common names, and as such will have the issues associated with the use of such names. They can also be the name of a ‘formula’, many of which are specified in a pharmacopoeia, for example Long Dan Xie Gan Wan. However, the ingredients of such formulae can vary, with one plant being substituted for another, and which plant is actually included in a particular product cannot be determined from the formula name alone. Lastly, in addition to the product and ingredient names, which may be regulated by the relevant medicines or food laws of a particular country, companies can also include trademarked or registered names on their products, such as Hyperiforce^®^.

### Relevance to *Hypericum perforatum*

Due to the nature of the references included in the MPNS Resource and its intended scope and audience, the MPNS Resource does not use the category ‘trade name’ and any that are included are categorised as common names.

## Discussion

*Hypericum* is a genus of over 500 species widely distributed in both the Old and New World. *Hypericum perforatum* L. is thought to have derived from a cross between *H. maculatum* Crantz subsp. *immaculatum* (Murb.) A.Fröhl. and *H. attenuatum* Fisch. ex Choisy where their distributions likely overlapped in western Siberia. Caution is advised when identifying plant material to be used for research or in medicinal products or food supplements/botanicals. Brief details of the variation between the subspecies of *H. perforatum* have been included, but Robson^16^ should be consulted for full descriptions and distributions, of *H. perforatum*,* H. maculatum*,* H. attenuatum*, their infraspecific taxa and hybrids and details of how they differ from one another morphologically.

In general, there is consistency in the medical literature in the use of one scientific name for *H. perforatum* L., and that name is usually spelt correctly and in full. In addition, *H. perforatum* is not one of the 4% of binomials for which homonyms exist as the binomial was only published once, by Carl Linnaeus. The chances of miscommunication about this species are therefore minimised, and issues associated with the use of synonyms and incorrect or absent plant name authors are not currently relevant to this species. For these reasons, we can conclude that communication in the medicinal literature with regard to *H. perforatum* is largely effective when its scientific name is included, which is not the case for many other medicinal plants.^5^ As bibliographic and other databases rarely employ a synonymised list of plant names, searching using synonyms as well as the accepted name will frequently increase the number of citations that are returned. This was shown to be the case with the example of *H. brasiliense*, demonstrating the importance of access to accurate and up‐to‐date taxonomy when investigating one of the many species from this medicinally important genus.

Although four subspecies are recognised for *H. perforatum*, all references in the MPNS Resource and the majority of literature in PubMed only refer to the species as a whole. The potential chemical variation within the species and across its distribution is rarely considered by researchers or regulators. The MPNS Resource displays the infraspecies of *H. perforatum*, a function which could highlight potential areas for further research and possible problems of mis‐ or imprecise identification.

Some published papers including one or more subspecies of *H. perforatum* identified by the PubMed searches suggest that there can be variation in the chemical composition both between the various subspecies, and between samples of the same subspecies. For example, using only one or two samples of each, Sagratini *et al*.^56^ and Maggi *et al*.^57^ investigated the concentrations of various constituents, including hyperforin and hypericin, in *H. perforatum* subsp. *perforatum* and subsp. *veronense*, as well as other *Hypericum* species; Maggi *et al*.^57^ referred to subsp. *veronense* by one of its synonyms, subsp. *angustifolium*. Other authors have concluded that one or other of the subspecies contains significantly higher concentrations of a purported active ingredient. Usai *et al*.^58^ found that an extract of *H. perforatum* subsp. *veronense* (as subsp. *angustifolium*) showed effective antidepressant activity in an animal model at a dose eight times lower than was required for a similar effect from subsp. *perforatum* and put this down to a higher hyperforin content. Finally, Dikmen *et al*.^59^ evaluated the wound healing potential of *H. perforatum* subsp. *perforatum* and subsp. *veronense*, concluding that they have different wound healing profiles. A significant factor for potential variability is thus being overlooked, and researchers are encouraged to not only include the species scientific name but also the subspecies wherever possible.

Over 80 unique non‐scientific names are used for *H. perforatum*. The most commonly used St John's wort can be applied to any species of the genus *Hypericum* and is thus potentially ambiguous, illustrating the imprecision of non‐scientific names. Despite the potential for confusion and miscommunication, we have found it to be common practice for research to be published using only non‐scientific names, and research on ‘St John's wort’ is no exception. Searching PubMed returned more citations for ‘St John's wort’ than for ‘*H. perforatum*’, illustrating the challenge of finding information about a plant, even for this species which is consistently referred to by a single scientific name.

The MPNS Resource includes six pharmaceutical names for *H. perforatum* and can be used to determine which one is used by a particular pharmacopoeia, and whether other plants are included in the definition – as is the case with ‘Herba hyperici’ in the Russian Pharmacopoeia. It is also the case that the meaning of a pharmaceutical name may differ between editions of the same pharmacopoeia as well as between one pharmacopoeia and another.

## Conclusions

As with all plants, it is important that natural product researchers working on *H. perforatum* are aware of all the names that have been used for it, and which are potentially ambiguous, so that they can undertake effective and comprehensive literature searches and data mining. To maximise the impact of their own publications, they should also be aware of and include the currently accepted scientific name and any scientific synonyms and non‐scientific names that are used in relevant regulations such as a particular pharmacopoeia.

Using unambiguous names when referring to medicinal plants is necessary to communicate clearly when sourcing raw material, publishing research or defining regulations. This paper shows that non‐scientific names, when used alone, are inappropriate and imprecise, as their meaning is not fixed according to international scientific principles. Scientific names provide the only means of ensuring precision and avoiding ambiguity. Their use, nevertheless, poses its own problems, and these have been described and the implications outlined.

To overcome the challenge of information retrieval posed by the multiple names in use for medicinal plants and to facilitate unambiguous communication, Kew developed the MPNS Resource. The US Food and Drug Administration (FDA), the EMA and other health regulators internationally have recently developed and adopted within their regulatory frameworks the International Standardisation Organisation's ‘Identification of Medicinal Products’ (IDMP).^60^ This provides a mechanism by which alternative terms used in different countries for the same product and the substances that it contains are linked by reference to unique identifiers. IDMP includes herbal substances, for which the scientific name of the plant and the part of the plant to be used are essential to the definition.^61^, ^62^ MPNS is providing two controlled vocabularies for use in this new standard for the scientific plant names (including synonyms in use in health regulations) and plant parts.

The usefulness of MPNS to researchers, regulators and others with an interest in medicinal plants was demonstrated for *H. perforatum* L. By mapping all names used for a plant onto a single botanical taxonomy, MPNS provides reliable and comprehensive access to the regulations and references that include a plant, regardless of the name that is used. The taxonomy used by the MPNS Resource derives from Kew's core taxonomic resources and recognises four subspecies for *H. perforatum*. These subspecies vary morphologically but literature searches demonstrated that plant material used in research is rarely identified below the level of species. This paper also demonstrates the need for further research into potential chemical variation between subspecies, and the pharmacological implications. Brief descriptions of morphological differences and distributions are therefore provided for the subspecies to assist with their identification.

New versions of the MPNS Resource will be released via the MPNS portal on an ongoing basis to reflect changes to our understanding of plant taxonomy and nomenclature. It will further supply through a web service the terminology required for compliance with the IDMP standard, recently adopted by major medicines regulators.

## Supporting information


**Table S1.** Medicinal Plant Names Services (MPNS) resource V6 content.
**Table S2.** Medicinal plant references in the MPNS resource V6 that cite *Hypericum perforatum*.
**Table S3.** Non‐scientific names associated with *Hypericum perforatum* L. in the MPNS resource, excluding pharmaceutical names.
