# Supplementary material for: A review of issues of nomenclature and taxonomy of Hypericum perforatum L. and Kew's Medicinal Plant Names Services
Source: J Pharm Pharmacol. 2017 Oct 16;71(1):4–14. doi: 10.1111/jphp.12831 (PMC6585798; doi:10.1111/jphp.12831)
Supplement: Supplementary file 1 — Table S1. Medicinal Plant Names Services (MPNS) resource V6 content. Table S2. Medicinal plant references in the MPNS resource V6 that cite Hypericum perforatum. Table S3. Non‐scientific names associated with Hypericum perforatum L. in the MPNS resource, excluding pharmaceutical names. [file JPHP-71-4-s001.pdf]

Issues of nomenclature and taxonomy of *Hypericum perforatum* L. and Kew's Medicinal Plant Names Services

Elizabeth Anne Dauncey (corresponding author)
Royal Botanic Gardens, Kew, Richmond, Surrey TW9 3AB, UK
+44 (0)20 8332 5716
e.dauncey@kew.org

Jason Thomas Whitley Irving
Royal Botanic Gardens, Kew, Richmond, Surrey TW9 3AB, UK
j.irving@kew.org

Robert Allkin
Royal Botanic Gardens, Kew, Richmond, Surrey TW9 3AB, UK
+44 (0)20 8332 5717
r.allkin@kew.org

Supplementary Tables

Supplementary Table A: Medicinal Plant Names Services (MPNS) resource V6 content

Medicinal Plant Names Services resource V6 was released online on 5 October 2016 and was accessed through the MPNS portal via the link on www.kew.org/mpns.

V6 contained:

117 references (full list available online - www.kew.org/kew-science/people-and-data/resources-and-databases/medicinal-plant-names-services/references)

18,481 plants cited as having a medicinal use in those references, of which:
17,450 are species and
1,031 are infraspecies (subspecies, varieties, formas)
These plants belong to 360 families

For these plants, the references incorporated employ:
51,047 scientific name records of which 22,309 are unique
101,135 non-scientific name records for 68,772 unique non-scientific names including:
2,325 unique pharmaceutical names
66,463 unique other names (common and drug names)

MPNS linked these names to:
228,111 scientific names derived from Kew's taxonomic databases.

In addition, the MPNS portal provides:
- Information on the part used (when this is given in a reference)
- Links out to other information sources using either the accepted scientific name, or the accepted scientific name + all known scientific synonyms (NCBI, EoL, WikiSpecies)

Supplementary Table B: Medicinal plant references in the MPNS resource V6 that cite *Hypericum perforatum*.

Abbreviation	Medicinal plant reference in full
Med. Pl. Trade Turkey (Özhatay et al., 1997)	Özhatay, N., Koyuncu, M., Atay, S. & Byfield, A. (1997). The Wild Medicinal Plant Trade in Turkey. Do�al Hayatı Koruma Derne�i, Istanbul.
Substances in Medicines, Australia (TGA, 2007)	Therapeutic Goods Administration (ed.) (2007). Substances that May be Used in Listed Medicines in Australia. Therapeutic Goods Administration, Symonston.
WHO Monographs Med. Pl. 2 (2002)	WHO (2004). WHO Monographs on Selected Medicinal Plants, Vol. 2. World Health Organization, Geneva
Archivos de Flora Iberica (Morales et al., 1996)	Morales, R., Macia, M.J., Dorda, E. & Garcia Villaraco, A. (1996). Archivos de Flora Iberica, Numero 7, Nombres Vulgares, 2.. Real Jardin Botanico CSIC, Madrid.
British Pharmacopoeia (2008)	Medicines and Healthcare Products Regulatory Agency (2011). British Pharmacopoeia 2012, Vol. 4. Stationery Office, London.
British Pharmacopoeia (2012)	Medicines and Healthcare Products Regulatory Agency (2014). British Pharmacopoeia 2012, Vol. 4. Stationery Office, London.
British Pharmacopoeia (2014)	Medicines and Healthcare Products Regulatory Agency (2008). British Pharmacopoeia 2009, Vol. 4. Stationery Office, London.
Demand & Supply of Med. Pl. In India (Ved et al., 2008)	Ved, D.K. & Goraya, G.S. (2008). Demand and Supply of Medicinal Plants in India. FRLHT, Bangalore.
Dizionario Delle Erbe Med. (Suozzi, 1995)	Suozzi, R.M. (1995). Dizionario Delle Erbe Medicinali: Oltre Duecentocinquanta Specie Vegetali Esaminate Nei Loro Effetti Curativi e Nella Loro Utilizzazione Practica. Newton Compton Editori, Rome.
EMA Community Monographs (2006 - 2014)	European Medicines Agency (2006-2014). Community Herbal Monographs (Selected Final Versions Adopted Between 2006 and 2014). European Medicines Agency (EMA)/The Committee on Herbal Medicinal Products (HMPC), s.loc.
Encyclopedia of Herb. Medicine (Bartram, 1995)	Bartram, T.H. (1995). Encyclopedia of Herbal Medicine. Grace Publishers, Christchurch, Dorset.
European Pharmacopoeia 6th edn. (2007)	European Directorate for the Quality of Medicines & Health Care (ed.) (2007). European Pharmacopoeia, Vol. 1-2 (6th edn). Council of Europe, Strasbourg.
European Pharmacopoeia, 7th edn. (2012)	European Directorate for the Quality of Medicines & Health Care (EDQM) (2012). European Pharmacopoeia, Pharmacopée Européenne (7.8 edn). Council of Europe, Strasbourg.
Farmacopea Argentina, 8th edn.	Anon. (2012). Farmacopea Argentina (8th edn). Vol. 3 (viewed: 09/09/2012). Ministerio de Salud, s.loc.
Farmacopea Herbolaria Mexicanos (2013)	Comisión Permanente de la Farmacopea de los Estados Unidos Mexicanos (ed.) (2013). Farmacopea Herbolaria de los Estados Unidos Mexicanos (2nd edn). Secretaria de Salud, Mexico City.
GRIN Report: World Economic Plants (Wiersema, 1999)	Wiersema, J.H. & Leon, B. (1999). World Economic Plants: A Standard Reference. CRC Press, Boca Raton, Florida. Retrieved as GRIN report on 17/03/2015 using query 'Medizin = Alle Nutzungen'.
Hagers Handbuch (Hänsel et al., 1992-1998)	Hänsel, R. et al. (1992-1998). Hagers Handbuch der Pharmazeutischen Praxis, Vol. 1-5 (5th edn). s.loc
Herbs of Commerce (McGuffin et al., 2000)	McGuffin, M., Kartesz, J.T., Leung, A.Y. & Tucker, A.O. (2000). Herbs of Commerce (2nd edn). AHPA, Silver Spring.
Kräuter Gewürze und Heilpflanzen (Bremness, 1994)	Bremness, L. (1994). Kräuter Gewürze und Heilpflanzen. Ravensburger.
Les Plantes Arom. et Med. (Bremness, 1996)	Bremness, L. (1996). Les Plantes Aromatiques et Médicinales: Le Guide Visuel de Plus de 700 Espèces Végétales à Travers Le Monde. Bordas, Paris.
Les Plantes Méd. de la Pharmacopée: Liste A (2000)	Anon. (2000). Les Plantes Méd. de la Pharmacopée (Liste A). Société Française d'Ethnopharmacologie, s.loc.
Med. Pl. Uzbekistan & Kyrgyzstan (Eisenman et al., 2013)	Eisenman, S.W., Zaurov, D.E. & Struwe, L. (ed.) (2013). Medicinal Plants of Central Asia: Uzbekistan and Kyrgyzstan. Springer, New York.
Med. Pl. of S. Africa (Arnold et al., 2002)	Arnold, T.H., Prentice, C.A., Hawker, L.C., Snyman, E.E., Tomalin, M., Crouch, N.R. & Pottas-Bircher, C. (2002). Medicinal and Magical Plants of Southern Africa: An Annotated Checklist. Strelitzia 13: 1-203.
Med. Pl. of the Russian Pharm. (Shikov, 2014)	Shikov, A.N. et al. (2014). Medicinal Plants of the Russian Pharmacopoeia: Their History and Applications. Journal of Ethnopharmacology. 154(3): 481-536, s.loc.
Med. Pl. of the World (Wyk & Wink, 2004)	Wyk, B. van & Wink, M. (2004). Medicinal Plants of the World: An Illustrated Scientific Guide to Important Medicinal Plants and their Uses. Timber Press, Portland, Oregon.
Native American Ethnobotany (Moerman, 1998)	Moerman, D.E. (1998). Native American Ethnobotany. Timber Press, Portland, Oregon.

Native Med. Plants of Mt. Rtanj (Zlatković, 2014)	Zlatković, B.K. et al. (2014). Traditional Use of the Native Medicinal Plant Resource of Mt. Rtanj (Eastern Serbia): Ethnobotanical Evaluation and Comparison. Journal of Ethnopharmacology. 151.1(2014): 704-713
Pharmacopoeia Helvetica (Swissmedic, 2006)	Swissmedic (ed.) (2006). Pharmacopoeia Helvetica: Deutsche Ausgabe (10th edn). Swissmedic, Bern.
Pharmacopoeia of China (2005)	Chinese Pharmacopoeia Commission (ed.) (2005). Pharmacopoeia of the People's Republic of China 2005. People's Medical Publishing House, Beijing.
Pharmacopoeia of China (2010)	Chinese Pharmacopoeia Commission (ed.) (2010). Pharmacopoeia of the People's Republic of China 2010. People's Medical Publishing House, Beijing.
Pharmacopoeia of China (2015)	China Pharmacopoeia Commission (ed.) (2015). Pharmacopoeia of the People's Republic of China 2015, Chinese edition. China Medical Science and Technology Press, Beijing.
Pl. Med. Totius Mundi (Penso & Proserpio, 1997)	Penso, G. & Proserpio, G. (1997). Index Plantarum Medicinalium Totius Mundi eorumque Synonymorum (2nd edn). OEMF, Milano.
Plants of Econ. Importance (FAO, 1983)	FAO (1983). Plants and Plant Products of Economic Importance. Food and Agriculture Organization of the United Nations Terminology Bulletin 25/1. Rome.
Timber Press Plant Names (Erhardt et al., 2009)	Erhardt, W. von, Götz, E., Bödeker, N. & Seybold, S.; Coombes, A. (ed.) (2009). The Timber Press Dictionary of Plant Names. Timber Press, Portland, Oregon.
U.S. Homeopathic Pharmacopoeia	HPUS (2009). HPUS Online Database (viewed: 26/10/2009). Homoeopathic Pharmacopoeia of the United States (HPUS)
U.S. Pharmacopoeia USP 37 (2013)	United States Pharmacopeial Convention (ed.) (2013). The United States Pharmacopeia USP 37. The National Formulary NF 32. 2014. United States Pharmacopeial Convention, Rockville.
U.S. Pharmacopoeia USP 32 (2008)	United States Pharmacopeial Convention (ed.) (2008). The United States Pharmacopeia USP 32. The National Formulary NF 27. 2009, Vol. 1-3. United States Pharmacopeial Convention, Rockville.

Supplementary Table C: Non-scientific names associated with *Hypericum perforatum* L. in the MPNS resource, excluding pharmaceutical names.

Non-scientific name	Part of plant used medicinally	Reference abbreviation
äkta johannesört		GRIN Report: World Economic Plants (Wiersema, 1999)
Balsana	flower, aerial parts	WHO Monographs Med. Pl. 2 (2002)
bassan	flower, aerial parts	WHO Monographs Med. Pl. 2 (2002)
bossant	flower, aerial parts	WHO Monographs Med. Pl. 2 (2002)
Cacciadiavoli		Dizionario Delle Erbe Med. (Suozzi, 1995)
common St John's Wort	flower, aerial parts	WHO Monographs Med. Pl. 2 (2002)
Common St. John's Wort		Native American Ethnobotany (Moerman, 1998)
Common St. John's wort Herb	aerial parts	Pharmacopoeia of China (2010)
Common St. John'swort Herb	aerial parts	Pharmacopoeia of China (2005)
Common St. Johnswort	herb	Med. Pl. Uzbekistan & Kyrgyzstan (Eisenman et al., 2013)
corazoncillo	flower, aerial parts	WHO Monographs Med. Pl. 2 (2002)
dendlu	flower, aerial parts	WHO Monographs Med. Pl. 2 (2002)
devil's scourge	flower, aerial parts	WHO Monographs Med. Pl. 2 (2002)
Echtes Johanniskraut	herb	Med. Pl. of the World (Wyk & Wink, 2004)
echtes Johanniskraut	flower, aerial parts	WHO Monographs Med. Pl. 2 (2002)
Eisenblut	flower, aerial parts	WHO Monographs Med. Pl. 2 (2002)
erba di San Giovanni	herb	Med. Pl. of the World (Wyk & Wink, 2004)
erba di San Giovanni	flower, aerial parts	WHO Monographs Med. Pl. 2 (2002)
flor de sao joao	flower, aerial parts	WHO Monographs Med. Pl. 2 (2002)
Frische Johanniskraut-Triebspitzen	stem tips	Pharmacopoeia Helvetica (Swissmedic, 2006)
fuga daemonum	flower, aerial parts	WHO Monographs Med. Pl. 2 (2002)
goat weed	herb	Med. Pl. Uzbekistan & Kyrgyzstan (Eisenman et al., 2013)
goatweed		GRIN Report: World Economic Plants (Wiersema, 1999)
guan ye lian qiao		GRIN Report: World Economic Plants (Wiersema, 1999)
Guanyejinsitao	aerial parts	Pharmacopoeia of China (2005)
Guanyejinsitao	aerial parts	Pharmacopoeia of China (2010)
Guanyejinsitao		Pharmacopoeia of China (2015)
hardhay	flower, aerial parts	WHO Monographs Med. Pl. 2 (2002)
Hartheu	flower, aerial parts	WHO Monographs Med. Pl. 2 (2002)
herbe à mille trous	flower, aerial parts	WHO Monographs Med. Pl. 2 (2002)
Herbe da la St. Jean		Plants of Econ. Importance (FAO, 1983)
Herbe da la St. Jean		Timber Press Plant Names (Erhardt et al., 2009)

herbe de millepertuis	flower, aerial parts	WHO Monographs Med. Pl. 2 (2002)
Herrgottsblut	flower, aerial parts	WHO Monographs Med. Pl. 2 (2002)
Hexenkraut	flower, aerial parts	WHO Monographs Med. Pl. 2 (2002)
Hierba de San Juan		Archivos de Flora Iberica (Morales et al., 1996)
Hierba de San Juan		Encyclopedia of Herb. Medicine (Bartram, 1995)
hierba de San Juan	flower, aerial parts	WHO Monographs Med. Pl. 2 (2002)
Hierba de San Juan, parte aérea	sumidad florida seca, o las partes aéreas, enteras o fragmentadas	Farmacopea Herbolaria Mexicanos (2013)
hiperico	flower, aerial parts	WHO Monographs Med. Pl. 2 (2002)
Hipérico, hierba	aerial parts	Farmacopea Argentina, 8th edn.
hipericon	flower, aerial parts	WHO Monographs Med. Pl. 2 (2002)
Hipéricón		Archivos de Flora Iberica (Morales et al., 1996)
houfarighoun	flower, aerial parts	WHO Monographs Med. Pl. 2 (2002)
Hypericum	flowering top	British Pharmacopoeia (2014)
Hypericum for Homoeopathic Preparations		British Pharmacopoeia (2012)
Hypericum for Homoeopathic Preparations	flowering plant	British Pharmacopoeia (2014)
Hypericum for homoeopathic preparations	flowering plant	European Pharmacopoeia 6th edn. (2007)
Hypericum for homoeopathic preparations	whole plant	European Pharmacopoeia, 7th edn. (2012)
Hypericum perforatum		U.S. Homeopathic Pharmacopoeia
Iperico		Dizionario Delle Erbe Med. (Suozzi, 1995)
iperico	herb	Med. Pl. of the World (Wyk & Wink, 2004)
iperico	flower, aerial parts	WHO Monographs Med. Pl. 2 (2002)
Jageteufel	flower, aerial parts	WHO Monographs Med. Pl. 2 (2002)
Johanneskruid		GRIN Report: World Economic Plants (Wiersema, 1999)
Johannisblut	flower, aerial parts	WHO Monographs Med. Pl. 2 (2002)
Johanniskraut		Kräuter Gewürze und Heilpflanzen (Bremness, 1994)
Johanniskraut		Plants of Econ. Importance (FAO, 1983)
Johanniskraut		Timber Press Plant Names (Erhardt et al., 2009)
Johanniskraut	flower, aerial parts	WHO Monographs Med. Pl. 2 (2002)
John’s wort	flower, aerial parts	WHO Monographs Med. Pl. 2 (2002)
Jottannesort	flower, aerial parts	WHO Monographs Med. Pl. 2 (2002)
Kantarion	Herba	Native Med. Plants of Mt. Rtanj (Zlatković, 2014)
Kizil-poicha	herb	Med. Pl. Uzbekistan & Kyrgyzstan (Eisenman et al., 2013)
Klamath weed	herb	Med. Pl. Uzbekistan & Kyrgyzstan (Eisenman et al., 2013)
klamath weed	flower, aerial parts	WHO Monographs Med. Pl. 2 (2002)
Klamathweed		GRIN Report: World Economic Plants (Wiersema, 1999)
Konradskraut	flower, aerial parts	WHO Monographs Med. Pl. 2 (2002)
Kozonokcholuu sary chay chop	herb	Med. Pl. Uzbekistan & Kyrgyzstan (Eisenman et al., 2013)
Liebeskraut	flower, aerial parts	WHO Monographs Med. Pl. 2 (2002)
Lord God’s wonder plant	flower, aerial parts	WHO Monographs Med. Pl. 2 (2002)
Mannskraft	flower, aerial parts	WHO Monographs Med. Pl. 2 (2002)
Millepertuis		Les Plantes Arom. et Med. (Bremness, 1996)
millepertuis	flowering tops	Les Plantes Méd. de la Pharmacopée: Liste A (2000)
Millepertuis		Plants of Econ. Importance (FAO, 1983)
Millepertuis		Timber Press Plant Names (Erhardt et al., 2009)
millepertuis	flower, aerial parts	WHO Monographs Med. Pl. 2 (2002)
millepertuis perforé	herb	Med. Pl. of the World (Wyk & Wink, 2004)
pelicao	flower, aerial parts	WHO Monographs Med. Pl. 2 (2002)
perforata	flower, aerial parts	WHO Monographs Med. Pl. 2 (2002)
perforate St John’s wort	flower, aerial parts	WHO Monographs Med. Pl. 2 (2002)
perforate St. John's wort	herb	Med. Pl. of the World (Wyk & Wink, 2004)
perforate St. John's-wort		GRIN Report: World Economic Plants (Wiersema, 1999)
Pilatro		Dizionario Delle Erbe Med. (Suozzi, 1995)
pinillo deoro	flower, aerial parts	WHO Monographs Med. Pl. 2 (2002)
quian-ceng lou	flower, aerial parts	WHO Monographs Med. Pl. 2 (2002)
racecourseweed		GRIN Report: World Economic Plants (Wiersema, 1999)
sarı kantaron	vegetative parts	Med. Pl. Trade Turkey (Özhatay et al., 1997)
seiyouotogiri	flower, aerial parts	WHO Monographs Med. Pl. 2 (2002)
sint janskruid	flower, aerial parts	WHO Monographs Med. Pl. 2 (2002)
St Jan’s kraut	flower, aerial parts	WHO Monographs Med. Pl. 2 (2002)
St John’s Wort	flower, aerial parts	WHO Monographs Med. Pl. 2 (2002)
St. John's wart	Flower	Demand & Supply of Med. Pl. In India (Ved et al., 2008)
St. John's Wort	flowering top	British Pharmacopoeia (2008)

St. John's Wort		British Pharmacopoeia (2012)
St. John's Wort	flowering top	British Pharmacopoeia (2014)
St. John's wort	herba	EMA Community Monographs (2006 - 2014)
St. John's wort	herba	EMA Community Monographs (2006 - 2014)
St. John's wort	flower	European Pharmacopoeia 6th edn. (2007)
St. John's wort	herb	Med. Pl. of the World (Wyk & Wink, 2004)
St. John's Wort	flowering tops or aerial parts	U.S. Pharmacopoeia USP 32 (2008)
St. John's-wort		GRIN Report: World Economic Plants (Wiersema, 1999)
St. John‘s wort		Herbs of Commerce (McGuffin et al., 2000)
St. John’s wort	flowering top	European Pharmacopoeia, 7th edn. (2012)
St. John’s Wort	flowering top, aerial parts	U.S. Pharmacopoeia USP 37 (2013)
St. John`s Wort		Encyclopedia of Herb. Medicine (Bartram, 1995)
St. John`s Wort		Plants of Econ. Importance (FAO, 1983)
St. John`s Wort		Timber Press Plant Names (Erhardt et al., 2009)
St. Johnswort	herb	Med. Pl. Uzbekistan & Kyrgyzstan (Eisenman et al., 2013)
tenturotou	flower, aerial parts	WHO Monographs Med. Pl. 2 (2002)
Teufelsflucht	flower, aerial parts	WHO Monographs Med. Pl. 2 (2002)
Tiptonweed		GRIN Report: World Economic Plants (Wiersema, 1999)
Tüpfel-Hartheu		GRIN Report: World Economic Plants (Wiersema, 1999)
Tüpfel-Johanniskraut		GRIN Report: World Economic Plants (Wiersema, 1999)
Tüpfel-Johanniskraut	herb	Med. Pl. of the World (Wyk & Wink, 2004)
Tüpfelhartheu	flower, aerial parts	WHO Monographs Med. Pl. 2 (2002)
witches’s herb	flower, aerial parts	WHO Monographs Med. Pl. 2 (2002)
zveroboj obyknovenny		GRIN Report: World Economic Plants (Wiersema, 1999)
Zveroboy prodyryavlennyy	herb	Med. Pl. Uzbekistan & Kyrgyzstan (Eisenman et al., 2013)
zwieroboij	flower, aerial parts	WHO Monographs Med. Pl. 2 (2002)
